# Nurturing Sustainable Development: The Interplay of Parenting Styles and SDGs in Children’s Development

**DOI:** 10.3390/children11060695

**Published:** 2024-06-06

**Authors:** Cristina Tripon

**Affiliations:** Teacher Training and Social Sciences Department, National University of Science and Technology Politehnica Bucharest, 060042 Bucharest, Romania; cristina.tripon@upb.ro

**Keywords:** SDGs, parenting styles, children development, families

## Abstract

This study delves into the dynamics of parenting styles and their impact on the cognitive and social–affective development of children within diverse family populations, contextualized within the framework of Sustainable Development Goals (SDGs). Drawing from a sample population comprising families from various socio-economic backgrounds and cultural contexts, the research explores the nuanced interactions between parenting approaches—ranging from authoritarian/permissive to democratic—and children’s developmental trajectories. By examining families with different numbers of children, this study elucidates the differential effects of parenting styles on cognitive adaptability and social–emotional skills across varying family structures. Democratic parenting emerges as a critical factor in promoting children’s well-being, equitable access to education, and the fostering of peaceful societies, aligning with SDGs 3, 4, and 16. Furthermore, the research addresses disparities in child development outcomes, shedding light on issues of gender equality (SDG 5) and reduced inequalities (SDG 10) within the context of family dynamics. Through a comprehensive analysis of diverse family populations, this study underscores the significance of inclusive and nurturing parenting practices in advancing sustainable development objectives, advocating for collaborative efforts (SDG 17) to support families in fostering optimal child development outcomes for all.

## 1. Introduction

The Sustainable Development Goals (SDGs) are a collection of 17 interlinked global goals designed to be a “blueprint to achieve a better and more sustainable future for all”. Adopted by the United Nations in 2015, the SDGs are intended to be achieved by the year 2030. The SDGs cover a wide range of social and economic development issues, including poverty, hunger, health, education, climate change, gender equality, water, sanitation, energy, urbanization, environment, and social justice [1]. The goals are interconnected—often, the key to success with one will involve tackling issues more commonly associated with another.

The SDGs build on the success of the Millennium Development Goals (MDGs), which were agreed upon in 2000 and were aimed at an array of issues that included reducing extreme poverty rates, stopping the spread of HIV/AIDS, and providing universal primary education, all by the target date of 2015 [2]. The SDGs go much further, addressing the root causes of poverty and the universal need for development that works for all people.

Achieving the SDGs requires the partnership of governments, the private sector, civil society, and citizens alike to make the vision a reality. They provide a shared blueprint and framework for all countries and all stakeholders to work together to build a better world [3,4]. The SDGs represent an ambitious and comprehensive agenda, addressing the interconnected economic, social, and environmental dimensions of sustainable development.

Parenting styles play a crucial role in shaping the social–emotional development of students. Authoritarian, permissive, and democratic parenting styles represent distinct approaches, with varying impacts on children’s behavior and well-being. Understanding these differences is not only important for individual child development but also intersects with broader societal goals outlined in the United Nations’ Sustainable Development Goals (SDGs). This essay explores the relationship between parenting styles and the SDGs, focusing on the hypothesis that there are significant differences in social–emotional behavior between preschoolers exposed to authoritarian/permissive versus democratic parenting styles.

## 2. Parenting Styles

Parenting styles encompass various approaches that parents employ in raising their children [5,6,7,8]. These styles significantly impact children’s emotional development and behavior. Four main parenting styles are commonly recognized: democratic, authoritarian, permissive, and uninvolved.

The democratic style, characterized by high control and warmth, is associated with positive outcomes, such as lower depression rates and reduced substance abuse. In contrast, authoritarian parenting, marked by high control and low warmth, can increase the risk of depression and substance abuse in children. Permissive parenting, whether loving or uninvolved, can lead to issues like aggressive behavior and depressiveness. Understanding these parenting styles is crucial for promoting healthy child development and preventing negative outcomes. The democratic parenting style significantly impacts children’s self-esteem by fostering positive outcomes in various aspects of their lives. Research has consistently shown that democratic parenting leads to higher levels of self-esteem in children, promoting socially valued traits, democratic values, and discipline [9]. Additionally, democratic parenting has been found to increase life satisfaction, well-being, and academic performance in adolescents through the enhancement of self-esteem [10]. Studies have also indicated a strong correlation between democratic parenting and improved self-esteem among male pupils in primary schools, with a direct positive influence on their self-esteem levels [11]. Furthermore, exploring the parenting styles of mothers has revealed a democratic approach that contributes to high self-esteem in young children, emphasizing the importance of this style in child-rearing practices [12].

Democratic parenting is characterized by high levels of parental warmth, responsiveness, and support, combined with appropriate levels of control and expectations [10,12]. This parenting style encourages independence, values discipline, and fosters social and cognitive competence in children, leading to better outcomes in terms of their overall development [10]. Research indicates that democratic parents create an environment where children feel secure, respected, and understood, promoting the development of self-esteem, social skills, and democratic values [13]. Additionally, democratic parenting is associated with higher levels of negative mood regulation expectancies (NMREs) in adults, indicating their confidence in alleviating emotional distress, and fewer symptoms of depression, anxiety, and somatic issues [14].

Authoritarian parenting is a strict, high-demand, and low-responsiveness approach characterized by a strong emphasis on obedience, discipline, and control. Parents who adopt this style prioritize structure and rules over nurturing and open communication, often resulting in a rigid and regimented family environment. The impact of authoritarian parenting on children can be profound and long-lasting, influencing various aspects of their development and well-being. Children raised by authoritarian parents often experience higher levels of anxiety, depression, and low self-esteem. The lack of emotional support and constant pressure to meet high standards can lead to feelings of inadequacy and worthlessness [15]. Due to limited opportunities for open communication and self-expression, these children may struggle with social interactions. They might have difficulty forming and maintaining healthy relationships, often exhibiting lower social competence and empathy. While some children might become excessively compliant and dependent on authority figures, others might rebel against the strict controls imposed by their parents. This rebellion can manifest as defiance, aggression, or other behavioral problems [16]. Authoritarian parenting can have mixed effects on academic performance. While some children may excel due to the high expectations, others might underperform due to stress and a lack of intrinsic motivation. The external pressure to succeed can diminish their natural curiosity and love for learning [17]. Children from authoritarian homes often struggle with decision making and independence. They may lack confidence in their abilities and rely heavily on external validation and direction, hindering their development of autonomy and self-regulation [18].

Permissive parenting is characterized by a lack of structure and rules, inconsistency in discipline, excessive gift giving, and a tendency to avoid conflict while prioritizing friendship over authority [19]. This parenting style often leads to children being difficult to discipline, selfish, lacking manners, disrespectful, lazy, and impatient due to the absence of clear boundaries and consequences set by parents [20]. Research has shown that permissive parenting can have negative impacts on children’s emotional development, leading to issues, such as anger expression, defiance, and difficulty in emotional regulation [12]. Additionally, studies have linked permissive parenting to poor academic achievement, decreased psychological health, lower quality of life, and an increased risk of juvenile delinquency among adolescents [21]. Despite some debates on the severity of its effects, permissive parenting has been associated with adverse outcomes in children’s behavior and well-being, emphasizing the importance of balanced and democratic parenting approaches for healthy child development.

The uninvolved parenting style, characterized by low sensitivity and low demand from parents towards their children, has been associated with negative outcomes such as higher depressiveness in Asian females compared to African-American females [22]. Research indicates that this style is a strong negative predictor of emotional involvement in teaching and can lead to low levels of cognitive and social involvement in students [23]. Additionally, the uninvolved parenting style has been linked to an increased risk of depression and substance abuse in children and adolescents, highlighting the detrimental effects of this approach on mental health and well-being [24]. Understanding the impact of parenting styles, including the uninvolved style, is crucial in promoting positive developmental outcomes and enhancing children’s overall well-being [25].

Parenting styles significantly impact children’s development and long-term well-being. Understanding the different styles—democratic, authoritarian, permissive, and uninvolved—allows parents to make informed choices that promote positive outcomes for their children. By emphasizing a balance of responsiveness and demandingness, parents can foster an environment that supports their children’s growth, independence, and overall happiness.

## 3. Intersection with Sustainable Development Goals

Parenting styles can influence children’s socio-emotional development, which, in turn, can impact their future socio-economic status. Research [26,27] suggests that children raised in democratic households tend to exhibit better self-regulation and interpersonal skills, which are vital for socio-economic success. By promoting democratic parenting practices that nurture children’s emotional intelligence and resilience, societies can contribute to breaking the cycle of poverty and advancing the goals of SDG 1.

The social–emotional well-being of children is fundamental to achieving SDG 3, which aims to ensure healthy lives and promote well-being for all ages. Research [28,29] suggests that children raised in democratic parenting environments tend to exhibit better emotional regulation, interpersonal skills, and mental health outcomes compared to those in authoritarian or permissive environments. By fostering a nurturing and supportive atmosphere, democratic parenting contributes to the holistic well-being of children, aligning with SDG 3.

Democratic parenting encourages children to express and regulate their emotions in a supportive environment. When children learn to understand and manage their emotions effectively, they are better equipped to cope with stress and adversity, leading to improved mental well-being and overall health outcomes [30]. This aligns with SDG 3’s objective of promoting emotional well-being as a crucial component of healthy lives.

In democratic parenting environments, children are encouraged to communicate openly, empathize with others, and resolve conflicts constructively. These interpersonal skills are essential for building positive relationships, fostering social support networks, and reducing the risk of social isolation or loneliness, all of which contribute to improved mental health and well-being [31].

Research [32,33] consistently shows that children raised in democratic households have lower levels of anxiety, depression, and behavioral problems compared to those in authoritarian or permissive environments. By providing a nurturing and supportive atmosphere where children feel valued, respected, and heard, democratic parenting helps mitigate the risk factors associated with poor mental health, thereby promoting overall well-being and aligning with the objectives of SDG 3.

The social–emotional well-being of children has long-term implications for their physical health and longevity. Positive social relationships, emotional resilience, and effective stress management skills acquired through democratic parenting contribute to healthier lifestyle choices, reduced risk of chronic diseases, and increased life satisfaction in adulthood [34], thus supporting the overarching goal of SDG 3 to ensure healthy lives and well-being for all ages.

Children who develop strong social–emotional skills in democratic parenting environments are less likely to engage in risky behaviors, such as substance abuse, violence, or unsafe sexual practices [35]. By promoting healthy decision making, self-esteem, and peer relationships, democratic parenting acts as a protective factor against various health risks, ultimately contributing to the prevention of diseases and injuries, as outlined in SDG 3.

Democratic parenting fosters an inclusive and equitable environment where children from diverse backgrounds feel valued and respected. By addressing the unique emotional needs of each child and promoting a sense of belonging, democratic parenting contributes to reducing disparities in health outcomes across different population groups [26,34], thereby advancing the principle of leaving no one behind, a core tenet of SDG 3.

SDG 4 emphasizes inclusive and equitable quality education for all. Parenting styles influence children’s cognitive development, school readiness, and academic performance. Schoolers raised in democratic households, characterized by open communication, autonomy support, and collaborative decision making, often demonstrate higher levels of school engagement and academic achievement [36]. Thus, promoting democratic parenting practices can contribute to realizing the objectives of SDG 4 by fostering a conducive learning environment and enhancing educational outcomes for all children.

Democratic parenting encourages children to ask questions, explore their interests, and engage in critical thinking, which are essential components of cognitive development. By promoting intellectual curiosity and problem-solving skills from an early age, democratic parenting lays the foundation for lifelong learning and academic success [37,38], thus aligning with SDG 4’s objective of ensuring quality education for all.

Schoolers raised in democratic households are more likely to develop the social, emotional, and cognitive skills necessary for a smooth transition to formal schooling. By fostering autonomy, self-regulation, and collaboration, democratic parenting enhances children’s readiness to engage in classroom activities, follow instructions, and interact positively with peers and teachers [39], thereby supporting SDG 4’s goal of inclusive and equitable education.

Research [40,41,42] consistently demonstrates that children raised in democratic parenting environments exhibit higher levels of academic achievement compared to those in authoritarian or permissive households. By promoting a supportive learning environment characterized by open communication, encouragement, and shared decision making, democratic parenting enhances children’s motivation, self-esteem, and academic performance, contributing to the realization of SDG 4’s objectives.

Democratic parenting practices prioritize equity and inclusivity by valuing each child’s unique abilities, interests, and learning styles. By fostering a sense of belonging and acceptance within the family, democratic parenting lays the groundwork for promoting diversity, tolerance, and respect in educational settings [43,44]. This inclusive approach to parenting helps create a conducive learning environment where all children, regardless of background or ability, can thrive academically, thus advancing the goals of SDG 4.

Schoolers raised in democratic households tend to have positive attitudes towards authority figures and develop healthy relationships with teachers [38]. By promoting autonomy support, respect for others’ perspectives, and collaborative problem solving, democratic parenting fosters a sense of trust and mutual respect between students and educators [45]. These positive teacher–student relationships are essential for creating a supportive learning environment that enhances students’ academic engagement and achievement, in line with the objectives of SDG 4.

Democratic parenting instills lifelong learning skills, such as critical thinking, communication, and adaptability, which are essential for success in the 21st-century knowledge economy [46]. By encouraging children to take ownership of their learning, set goals, and seek out new experiences, democratic parenting promotes a growth mindset and a love for learning that extends beyond the classroom. These skills are invaluable for navigating the complexities of the modern world and contributing to sustainable development, aligning with the overarching goals of SDG 4.

Parenting styles may also influence the development of gender attitudes and roles in children. Democratic parenting, which promotes equality, respect, and shared decision making, has the potential to challenge traditional gender stereotypes and promote gender equality within families. By encouraging both boys and girls to express emotions, communicate openly, and participate in decision-making processes [47], democratic parenting contributes to breaking down gender barriers and advancing the goals of SDG 5.

Parenting styles play a crucial role in shaping children’s attitudes towards authority, conflict resolution, and respect for others’ rights. Democratic parenting, characterized by open communication and mutual respect, fosters a culture of dialogue, empathy, and cooperation within families [34,36,42]. By promoting democratic parenting practices, societies can instill values of tolerance, non-violence, and inclusivity in future generations, thus contributing to the objectives of SDG 16 aimed at promoting peaceful and inclusive societies.

## 4. Research Methodology

Our general hypothesis is as follows: Parenting style is assumed to influence children’s psychological development.

Our specific hypotheses are as follows:

**H1:**  *It is assumed that there are significant differences in cognitive behaviors between children whose parents adopt an authoritarian/permissive parenting style and children whose parents adopt a democratic parenting style.*

**H2:**  *Significant differences in social–affective behavior are assumed to exist between children whose parents adopt the authoritarian/permissive parenting style and children whose parents adopt the democratic parenting style.*

### 4.1. Participants

This research involved children (N = 171) aged between 5 years and 6 years and 2 months, from Bucharest. They were grouped into six groups, according to the parenting styles adopted by their own parents. Also, the research included parents of the evaluated children, aged 31–35 years, N = 171 dyads (mother–father). Initially 440 parents were surveyed, but following the construction of the participant batches based on the grouping variable, 342 parents, 171 mothers and 171 fathers, remained (171 dyads). Parents who obtained average scores after completing the Parenting Style questionnaire were excluded from the study. The groups of parents were constituted taking into account two criteria: the parenting style practiced and the consensus/disagreement between the parenting styles adopted by the parents. Each dyad was matched to its own child, the samples of parents and children being dependent.

Given the participants and the criteria for grouping, the groups were:

G1: permissive–permissive, N = 30 dyads ↔—G1’: children, N = 30; 16 girls, 14 boys.
G2: democratic–democratic, N = 30 dyads ↔—G2’: children, N = 30; 15 girls, 15 boys.
G3: authoritarian–authoritarian, N = 26 dyads ↔—G3’: children, N = 26; 12 girls, 14 boys.
G4: democratic–permissive, N = 30 dyads ↔—G4’: children, N = 30; 14 girls, 16 boys.
G5: democratic–authoritarian, N = 27 dyads ↔—G5’: children, N = 27; 12 girls, 13 boys.
G6: permissive–authoritarian, N = 28 dyads ↔—G6’: children, N = 28; 12 girls, 16 boys.
G8: children, N = 30, 15 girls and 15 boys. This last group was constituted by random sampling from the children population investigated in the present research.

### 4.2. Research Instruments

In order to investigate the level of the children’s psychological development, the two defining behavioral domains were considered: cognitive and social–affective.

The assessment of cognitive behaviors was carried out with the help of the scales for the assessment of the child’s psychological development, a combined instrument developed by the authors Ottenbreit and Dobson in 2004 [48] and Özge and collaborators [49]. (Strengths and Difficulties Questionnaire—SDQ).

In order to obtain a more complete knowledge of children’s psychological development and to capture particular aspects of individual psychological development, the characteristics of psychological normality were established, in relation to four areas of behavior: motor (I), cognitive (II), verbal (III), social–affective (IV) (detailed in Table 1, areas of behaviors, and Table 2, examples of items.

Each behavior corresponds to a certain number of items/tests. The child’s results are recorded on the individual score sheet. It should be noted that for testing the research hypotheses, items of cognitive behavior were included along with those of motor and verbal behavior. It is well known that motor skills underlie the development of cognition and that there is an inextricable link between language and thought. Thus, for the assessment of cognitive behavior, the scale comprised 16 items/probes. The raw scores obtained by each child were used for statistical interpretation, following the scoring procedure described above.

As the assessment of the level of development of social–emotional behavior was a particularly important aspect of the present research, we established the components of the social–affective behaviors, presented in Table 3, target dimensions of social–emotional behaviors, as well as the observational work carried out in the school environment.

The scale consists of 15 items. As with the scale used to assess cognitive behavior, raw scores were used for this scale.

In order to identify the parenting style adopted by each parent, the Parenting Style questionnaire was constructed. It is a two-dimensional questionnaire, developed on the basis of the authors’ theoretical model collected by Larzelere et all. [50]. Parenting style refers to the nature and characteristics of the family relationships in which the educational process takes place. The following working definition has been proposed: parenting style is the specific way in which parents relate to their children in their educational activities.

According to the basic theoretical model, two axes are taken into account in determining parenting style (Table 4—attachment dimension and control dimensions):

- Attachment axis, with the two extremes: rejection–attachment;
- Control axis, with the two extremes: permissiveness–restrictiveness.

**Table 4:** Attachment dimension and control dimensions.

Attachment Dimension	Control Dimension
Parental engagement in children’s activities; Support for children; Responsiveness to ‘younger generation’ issues; Relating emotional states to the child’s needs; Time allocated to the child’s education.	Imposing constraints on children’s activities; - Assignment of responsibilities; - Ways of exercising parental control; - Rigorous enforcement of parental control; - Control of compliance with the rules imposed.

Depending on the scores obtained on these, four parenting styles were established:

Permissive style (scores above average on attachment and scores below average on control);
Democratic style (above average scores in both dimensions);
Authoritarian style (above average scores on control and below average scores on attachment);
Neglect/rejection style (below average scores in both dimensions).

Taking these indicators into account, items were formulated for each dimension. The work with children and communication with parents were also taken into account.

Initially, 104 items were formulated. These were evaluated by 13 experts. The evaluation consisted of redistributing the items between the two dimensions and rating the wording of the items on a scale of 1 to 10 in terms of semantic characteristics. Following the evaluation, items with scores below and including 6 were eliminated, those with scores of 7 and 8 were reformulated, and those with scores of 9 and 10 were retained. Items that could not be classified in any dimension were also eliminated, as they were marked with “?” and due to inter-rater disagreement (in this respect, items classified in the same dimension by at least 8 out of 13 raters were kept).

For the pilot study, the questionnaire had 70 items (35/dimension), and α = 0.78 (N = 78) was obtained for the whole questionnaire. After eliminating items, the following coefficients were obtained: for attachment, α = 0.87 (N = 78), and for control, α = 0.88 (N = 78). The final form of the questionnaire comprises 46 items, 23/dimension. Responses are rated on a Likert scale in 5 steps: DT (strongly disagree), DP (partially disagree), N (neutral), AP (partially agree), TA (strongly agree). Each response variant is scored with 1, 2, 3, 4, 5p, respectively. These evaluations include exploratory and confirmatory factor analyses to determine the underlying factors, internal consistency assessments using Cronbach’s alpha, and tests for construct validity. To determine parenting style, the scores for each dimension are summed to see in which range of the scale/dimensions (set for both male and female) they fall. The scales for each dimension were constructed by gender, with the interpretation of each range being made according to the extremes of the two axes.

To describe parenting styles by both parents using a composite score, researchers often utilize questionnaires and rating scales that assess various dimensions of parenting behaviors (Table 5, parent dyad scores).

When embarking on research endeavors concerning parenting style and its potential impact on children’s cognitive and social–affective behaviors, the meticulous selection of assessment instruments is paramount. The chosen instruments must exhibit both reliability and validity, capable of capturing the intricate nuances inherent in parent–child dynamics and subsequent child developmental outcomes. In the context of the specific hypotheses posited, the instruments selected should harmonize with theoretical underpinnings regarding parenting styles and their anticipated influence on children’s cognitive and social–affective domains.

Commonly employed instruments for evaluating the parenting style mentioned above offer comprehensive assessments in various dimensions of parenting behaviors, including warmth, control, and autonomy granting, which are pivotal in delineating between authoritarian, permissive, and democratic parenting styles.

Theoretical frameworks furnish a robust foundation for comprehending the distinguishing features of each parenting style and their anticipated ramifications on child development. The authors of [51] delineate authoritarian parenting by its high control and low warmth, permissive parenting by low control and high warmth, and democratic parenting by a synthesis of high levels of both control and warmth. By aligning with these theoretical tenets, researchers can operationalize and evaluate the parenting styles envisaged to influence children’s cognitive and social–affective behaviors effectively.

In the realm of assessing children’s cognitive behaviors, instruments furnish objective measures of cognitive abilities, encompassing intelligence, problem-solving acumen, and linguistic proficiencies, facilitating the identification of potential disparities between children reared in authoritarian/permissive versus democratic parenting milieus.

Similarly, for the appraisal of children’s social–affective behaviors, instruments scrutinize diverse facets of social competence, emotional regulation, and behavioral adjustment, domains intricately entwined with parenting style. By leveraging established measures of social–affective behavior, researchers can effectively delineate the hypothesized variances between children nurtured in disparate parenting contexts.

### 4.3. Data Collection Procedures

The application of the research instruments acquired a specific character due to the age level of the participants involved in the research. The scales used to assess cognitive and social–affective behavior were applied individually to the children, the instruction of the samples being the same for all participants. The actual application took place in a familiar place for the child (the classroom), and objects that could distract the child’s attention were removed from the child’s field of vision. Children were tested after the construction of the parent groups. The technical material was prepared before each child entered the room; the examiner sat face to face with the child, and the furniture was age appropriate. The average duration of testing of each schooler was 30 min. The specific character of the application procedure also refers to what is called the “warming up period”, during which a kind of alliance is established between examiner and child. The purpose of this was to relax the child, to get to know the examiner, to familiarize him/her with the examiner’s presence. Usually, a conversation about the favorite toy or cartoon that was watched allowed the child to gain confidence in the examiner and relax very easily.

The assessment was exclusively in the form of a game, as the age of the children did not allow them to go through the tests in a mechanical, rigid manner. However, the examiner was clear about the aims of the test, adapting his style according to the specifics of the subject. The score obtained was noted on the child’s individual sheet, and on the reverse side, observations on the child’s social behavior and non-verbal communication were noted.

The scale to assess the social–emotional behavior of the children was given to the teachers of the groups to which the children belonged, respectively, to the referent, the instruction being noted on each copy. Teachers were asked to complete the scale for each child participating in the research. Thus, they were asked to rate with 1p or 0p each item on the given scale, as follows: with 1p, if the rated pupil often shows the behavior described by the item; with 0p, if the pupil does not show the behavior described by the item. At the end, the teachers summed up the scores given, noting the individual score in the box indicated.

The “Parenting Style” questionnaires were distributed to parents in envelopes, each with two copies: one for the mother and one for the father. The instructions were indicated on each questionnaire, explaining the purpose of completing the questionnaire and the need to write the child’s name. The confidentiality of the information obtained was also ensured, with parents having the right and opportunity to seal the envelopes when handing them in. The questionnaires (Table 6, validity and reliability of the instruments used) were handed out by both the examiner and the group educators. They were informed about the purpose of completing the questionnaire and its instruction. Questionnaires were initially distributed to 220 two-parent families. Following the establishment of the batches of participants in the actual research, 171 two-parent families participated.

## 5. Results

The results obtained from testing the first hypothesis are first presented in a descriptive form in the table below (Table 7, descriptive data—first hypothesis). It is double entry, with the means of the raw scores at 95% confidence intervals and the minimum and maximum scores recorded by the participants in each group listed horizontally. On the vertical side, the groups of parents established according to the style adopted and the consensus/contradiction between styles are listed.

The dependent variable is the cognitive behavior of children and is measured on an interval-report scale. This is because the raw scores obtained by each child on the Developmental Level Rating Scale for this behavioral domain were taken into account.

The independent variable, parenting style, is measured on a nominal scale, forming six groups of two-parent families.

To test Hypothesis 1, Unifactorial Analysis of Variance (ANOVA) was applied to see if there were significant differences between children in cognitive behavior imposed by the parenting style. Thus, the means of the raw scores obtained by the schoolchildren on the Cognitive Scale were compared. Unifactorial ANOVA revealed that there was no significant difference between the means of the school groups’ scores on cognitive behavior, even though the parents adopted different parenting styles in each group: F (5,165) = 0.52, *p* = 0.75 > 0.05. Given these results, the question arises “What is it that makes there no significant differences between levels of cognitive development?”. The answer will be given in the Discussion section.

**Figure 1:**
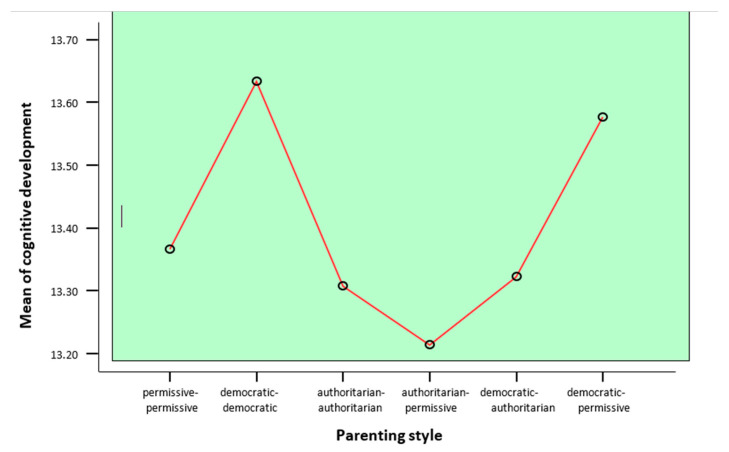
One-way analysis of variance (ANOVA)—Bonferroni.

The results obtained from testing the second hypothesis are first presented in a descriptive form in the table below (Table 8, descriptive data—second hypothesis and Figure 1). It is double entry, with the means of the raw scores at 95% confidence intervals and the minimum and maximum scores recorded by the participants in each group listed horizontally. On the vertical side, the groups of parents established according to the parenting style adopted and the consensus/contradiction between styles are listed.

For the test of Hypothesis 2, the Unifactorial Analysis of Variance (ANOVA) was also applied to see if there were significant differences between children in the social–affective behavior imposed by the parenting style. Thus, the means of the raw scores obtained by the children on the Social–affective Behavior Rating Scale were compared. This time, the Unifactorial ANOVA revealed a significant difference between the means of the scores of the groups of children on the level of social–affective behavior: F (5,165) = 35,881, sig. at α = 0.01.

Given the confirmation of the hypothesis, it can be said that parental style influences the social–affective behavior of children. The democratic style has a positive influence, materialized by the children obtaining significantly higher scores than those whose parents adopted the authoritarian/permissive style, the latter having a negative influence on the child’s social–affective behavior.

## 6. Discussion

Following the data collection and obtaining the results through statistical processing, a number of global and specific discussions and explanations are required. The purpose of these is to make sense of the whole research, to support the achievement of the proposed objectives and to provide a proper interpretation of what has been obtained from the hypothesis testing.

From the beginning of the description of the methodological approach in this research, the general hypothesis was launched: parenting style influences the psychological development of the child. The specific hypotheses were established on this basis. It is necessary to recall that the groups of participants were formed in a certain order. These references are made with respect to the groups of adults and children from a family background. The groups of parents—mother–father dyads—were established first, according to the parenting styles adopted by them and according to the consensus existing or not between parenting styles. Each dyad was assigned its own child, and six groups of children were formed, as was the number of parent groups.

Following the application of the One-way ANOVA, the first specific hypothesis of this research was refuted. The results showed no significant differences between the means of the groups of children. In other words, regardless of whether parents adopt different parenting styles compared to the styles of other parents and their child, there are non-significant differences between the levels of cognitive development. These can be explained by the interaction of fundamental developmental factors, as each child has his or her own hereditary endowment, having grown up and developed in a different family environment. As the statistical procedure itself provided an overview of the influence of parental style on a child’s cognitive development, the following explanations will also be comprehensive.

Attendance of kindergarten, before school, is considered a key factor for the present research, and it can be considered as the variable that, through its influence, determined effects on the dependent variable in this research. This led to the refutation of the first assumption. As stated, all the children involved in the research had attended kindergarten for at least two years. This explains why the refutation of the hypothesis cannot be considered a limitation. The results show the beneficial influence of the kindergarten environment, as teachers use the activities carried out with the children to develop their cognitive potential, develop skills, and form school-age skills. This aligns with the broader objectives of the Sustainable Development Goals (SDGs), particularly those related to education, health, and well-being.

The kindergarten environment plays a crucial role in laying the foundation for children’s cognitive development and school readiness. By providing a stimulating and supportive environment, teachers and parents can enhance children’s learning experiences, promote skill development, and prepare them for formal education [52]. This directly contributes to SDG 4, which aims to ensure inclusive and equitable quality education for all.

The positive impact of the kindergarten environment on children’s social, emotional, and physical well-being is evident in the sources. Outdoor education, nature-based activities, and social interactions in kindergartens contribute to children’s holistic development and mental well-being [53]. This focus on well-being aligns with multiple SDGs, including SDG 3 (Good Health and Well-being) and SDG 5 (Gender Equality), which emphasize the importance of mental health, social development, and equal opportunities for all.

An emphasis on teamwork, cooperation, self-confidence, and empathy in the kindergarten environment fosters positive social interactions and interpersonal skills among children [54]. These skills are essential for building inclusive and supportive communities, contributing to SDG 10 (Reduced Inequalities), which aims to reduce inequalities within and among countries.

By recognizing the critical role of the kindergarten environment in promoting children’s cognitive, social, and emotional development, educators and policymakers can work towards achieving the SDGs related to education, health, well-being, and social equality. Investing in high-quality early childhood environments that support children’s overall growth and learning is essential for building a sustainable and inclusive future in line with the principles of the SDGs.

The influence of education in the school and kindergarten environment can be positive, as long as there is unity between these and the family environment in terms of the demands placed on the child. Once the child enters kindergarten, the narrow family environment is overcome, and the new environment places new demands on the child, which are different from those of the family and which the child should respond to.

There is a tendency to make the most of the role of the kindergarten teacher, which can be encouraging or raise questions. First of all, it can be seen that, year after year, the work of the teacher is not only limited to working with children and collaborating with them. The evolving and complex role of educators in early childhood education emphasizes the need to engage with parents and align the influences from the family and nursery environments to ensure the child’s well-being. This can be linked to several Sustainable Development Goals (SDGs).

Educators need to explain their role in the preschool institution to parents, fostering a collaborative approach towards the common goal of the child’s well-being. Ensuring that the influences from the family and nursery environments are consistent and supportive contributes to creating a holistic educational experience for children, aligning with the goal of providing quality education for all [55]. This collaboration between parents and educators helps to address disparities in educational experiences and promotes inclusive and equitable access to quality early childhood education.

The child spends less time at school than at home, which is a more stable environment. The question is, however, how stable? The fact that the schoolchild spends more time at home than at nursery can sometimes be a disadvantage for the child, in the sense of losing the gains made in nursery. The family climate and parents’ upbringing can be disruptive factors that can confuse the child.

At school age, the educational influence of school is thought to increase in intensity until it becomes dominant. The results of testing the first hypothesis support this. The high scores obtained by children on the Cognitive Behavior Rating Scale also highlight the consensus between what the child requires, the school, and the family requirements. Conversations with teachers in the class to which the children assessed belonged confirmed this.

On the one hand, we can cite the parents’ awareness of the importance of attending kindergarten for the child’s psychological development; on the other hand, the adults’ total reliance on this environment, and on school work, can be considered, especially because of the professional overload they go through as adults.

Kindergarten attendance was, for the present research, the factor that was interposed between the psychological development of the older schoolchild and the parental parenting style, thus allowing us to express the idea that children do not differ significantly in terms of cognitive behavior, regardless of parenting styles.

It is noted, however, that the refutation of this hypothesis suggests the need for the child to enter the school or kindergarten environment as early as possible, which has an important constructive role.

The testing of the second hypothesis in this research revealed results that allowed for its acceptance, confirming the hypothesis. Given the data obtained, it can be said that the parenting style determines differences in the social–affective behavior of children. The results emphasize the significant impact of the parent–child relationship on a child’s social and emotional development, highlighting how this relationship influences the child’s behavior, interactions with adults and peers, adherence to rules, initiative, communication skills, and emotional expression. Moreover, they show their emotional feelings appropriately according to the situations in which they take part. A child who watches recreational programmers’ and does not laugh, or enjoys the displeasure of others, who is bored during modelling, mosaic, and household activities, considering them a burden and uninteresting, raises certain question marks in terms of his social–affective behavior.

The way parents interact with their children and the educational opportunities they provide can significantly impact a child’s social and emotional development, which are essential components of quality education. By fostering positive parent–child relationships that promote social skills, emotional intelligence, and effective communication, parents contribute to creating a supportive learning environment that enhances the child’s overall educational experience [56].

The results underscore the importance of emotional expression and appropriate social behavior, which are integral to mental well-being and healthy social relationships [57]. Parents play a crucial role in nurturing their children’s emotional intelligence and social skills, contributing to their overall well-being and mental health.

The development of skills, such as respecting rules, cooperating with others, and effectively communicating with peers and teachers, fosters a sense of social responsibility and contributes to building peaceful and inclusive societies [58,59]. By promoting positive social behaviors and emotional regulation in children, parents support the development of strong institutions based on respect, cooperation, and effective communication (SDG 16: Peace, Justice, and Strong Institutions).

Encouraging children to express their emotions appropriately and engage actively in social interactions helps break down gender stereotypes and promotes gender equality by fostering emotional intelligence and communication skills in all children, regardless of gender. Parents have a fundamental role to play in teaching children how to enjoy the moments of childhood that will be rich memories in adulthood. In the family, children grow and develop under the shaping action of the family environment. It is here that the child is introduced to the first patterns of relationships that will form the pattern of later relationships: they relate to the adult; the adult relates to them. It is an interaction whose positive or negative meanings are also determined by the parenting style (SDG 5, Gender Equality).

Permissive and authoritarian parenting styles have resulted in children achieving lower scores in social–emotional behavior than children whose parents adopted the democratic style. In general, children whose parents adopt the authoritarian style did not score on the items concerning relating to adults; preferring to play alone or watch TV; relating to teachers, in the sense of non-involvement in instructional–educational activities; expressive language in recreational activities, formulating answers to questions.

Unlike the girls who were described by the teachers as shy, distrustful, lacking courage, the boys were judged as lacking interest in educational activities, preferring aggressive games, often getting into conflicts with peers, taking their toys without asking permission. In terms of affective experiences, they often enjoy unpleasant events experienced by their peers.

Children whose parents adopted a permissive style did not score well on items concerning self-serving skills, sense of order, and relationships with peers. It should be pointed out that the differences between boys and girls are based on a purely qualitative criterion. This consists of teachers’ observations and analysis of the social–affective scales, completed by them. No statistical work has been carried out in this respect, as the research hypothesis is aimed at differences between children in general.

It was found that children whose parents are permissive show selfish behavior, do not give toys to their peers, are disinterested in educational activities, want to be informal leaders, are lazy when it comes to self-serving skills, often do not follow the rules of games, and want to impose their own “laws”. If others do not accept them, they withdraw but not for long, as they will resume their insistence.

Promoting democratic parenting not only enhances children’s immediate social–affective behavior but also contributes to long-term societal benefits, further aligning with multiple SDGs. For instance, SDG 3 focuses on ensuring healthy lives and promoting well-being for all ages. Children who experience positive social–affective development are more likely to enjoy better mental health, reduced stress levels, and overall well-being throughout their lives.

Furthermore, the positive effects of democratic parenting extend to SDG 5, which aims to achieve gender equality and empower all women and girls. Democratic parenting encourages equality and respect within the family unit, teaching children to value and practice gender equality from a young age. This foundational understanding can lead to more egalitarian attitudes and behaviors in adulthood, fostering environments where gender equality is respected and upheld.

In addition, democratic parenting supports SDG 16, which promotes peaceful and inclusive societies for sustainable development. By nurturing empathy, cooperation, and conflict resolution skills, democratic parenting helps children grow into adults who contribute positively to their communities, fostering peace and inclusivity.

To maximize the benefits of democratic parenting styles, it is crucial to provide parents with the necessary resources and education. This includes accessible parenting programs, community support groups, and educational materials that emphasize the importance of nurturing and supportive interactions. Governments, non-governmental organizations, and educational institutions should collaborate to promote these initiatives, ensuring that all parents, regardless of socioeconomic status, have the opportunity to adopt positive parenting practices.

By integrating democratic parenting strategies into broader social policies and educational frameworks, we can create a ripple effect that benefits individual families and society as a whole. This comprehensive approach will not only enhance the social–affective development of children but also contribute significantly to the achievement of the Sustainable Development Goals, ensuring a healthier, more equitable, and sustainable future for all.

Acceptance of the second hypothesis is a further argument for considering the impact of parenting style on children’s social and emotional behavior. The start of a harmonious development is given by the parents, the subsequent development of the child being influenced by the foundations laid in the family, hence the need for the foundations to be laid with attachment and control, with the parent striving to fulfil an important goal for their child’s life: balancing the two dimensions involved, attachment and control.

## 7. Conclusions

As the data show, children whose parents adopt the democratic style scored very well on the Social–Affective Behavior Rating Scale, demonstrating the positive influence of this balanced style on the child’s behavior. In general, these children know when and how to speak, are self-confident, have initiative in play, seek out the group of children, express their emotional feelings adequately, and are expressive in role playing and role playing. They relate to teachers very well, recognizing the authority they have earned.

Social and emotional behavior depends to a large extent on the establishment of fair relationships between family members. The way each parent relates to their child in their education is influenced by their own adult upbringing, educational level, values, attitudes and worldview. As shown in the first part, parents begin to question the effectiveness of the educational methods adopted when they encounter problems with their children.

The relational model provided by the family can be an inhibiting factor for the development of the child’s social–emotional behavior when it is authoritarian. The effects of the authoritarian style are soon felt, with the child becoming either introverted, shy, with low self-esteem, or aggressive, behaving as he or she saw fit, as others researchers [56] conclude.

Children whose parents have adopted an authoritarian style are clearly different from other groups, such as those whose parents are permissive.

In summary, the evidence suggests that by promoting democratic parenting practices that foster children’s emotional intelligence and resilience, societies can contribute to breaking the intergenerational cycle of poverty and advancing the objectives of the SDGs. This holistic approach to child development is crucial for creating more inclusive and sustainable communities.

## 8. Limitations

Research on the influence of parenting style on children’s social–affective behavior has yielded valuable insights, but it is not without limitations. One primary constraint is the complexity of categorizing parenting styles into distinct types, especially when considering nuanced combinations of behaviors. This research focuses on six primary dyads of parenting styles, and each of these can impact children’s social–affective development in unique ways, which complicates drawing clear, generalized conclusions. Further limitations of the research include the categorization of parenting styles: parents may exhibit a mix of styles, and their approach can vary based on the situation, the child’s behavior, and other contextual factors; cultural *differences*: parenting practices and their perceived effects can vary widely across different cultures; *measurement limitations*: research often relies on self-reported data from parents and children, which can be subject to biases such as social desirability bias or inaccurate recollection; individual differences among children: children are unique individuals with their own temperaments, personalities, and responses to their environments.

## Figures and Tables

**Table 1:** Areas of behaviors.

Motor Behavior (M)	Cognitive Behavior (C)	Verbal Behavior (V)	Social–Affective Behavior (S)
Balance in orthostasis	Identification of spatial positions, perception of object properties of length, size, weight, color	Acquisition of grammatical categories	Manifestations of independence, self-serving skills
Oculomotor coordination; General body-segment coordination	Representation activity; Temporal perception, notions of temporal orientation in acquisition	Speech, correct expression; Spoken and read language	Play activity; Relationship with adults and children

**Table 2:** Examples of items.

Instruments	Examples of Items
Cognitive domain	Walk correctly in a circle drawn on the floor. Draw a rhombus according to the pattern. Correct orientation movements in own body scheme with great ease. Makes a construction out of 10–12 cubes. Indicates and names 6–7 colors or shades. Identifies 3 main times of the day, reports common activities for these times. Knows and names 3–4 seasons. Lists 5–7 days of the week. Names from memory 4–5 general notions (clothing, furniture, vehicles, toys). Defines 4 objects or beings (ball, cat, coat, horse). Establishes similarities between 3 given notions (dog-cat; apple-pear; ice-cream) Count more than 10 cubes. Uses 3 adverbs of time correctly (today, tomorrow, yesterday). Tell more about 3 given images. Briefly tell a familiar story. Recognizes 2–3 letters correctly.
Socio-affective domains	Communicates easily with adults in the immediate environment. Gives up the toy at the request of another child. Enjoys role play. Respects the rules of the game in which he/she takes part. Arranges things neatly on his/her own. Participates actively in educational activities. Does not get into conflicts with other children. Is liked by peers. Reacts appropriately from an emotional point of view during recreational activities. Recites poems/stories in an expressive manner. Performs social tasks with pleasure. Dresses self, correctly. Takes initiative in organizing games. Greets and talks to adults in the immediate environment. Seeks out the group of children.

**Table 3:** Target dimensions of social–emotional behaviors.

Dimensions of Social–Emotional Behavior
Autonomy and initiative in relationships with adults, in play and other activities (instructional-educational and recreational); Relating to the learner and other adults in the immediate environment; Appropriate expression by the child of emotional-expressive behaviors according to the specific activities in which he/she is involved (watching theatre/circus performances, games–dramatizations, recitations, stories, singing); A sense of order in arranging personal belongings.

**Table 5:** Parent dyad scores.

Dimension	Scale	Female Standard	Male Standard	Interpretation
Attachment	1	36–52	36–62	Extreme rejection
	2	53–76	63–76	Moderate rejection
	3	77–89	77–86	Average scores
	4	90–105	87–99	Moderate attachment
	5	106–120	100–114	Extreme attachment
Control	1	36–53	26–42	Extreme permissiveness
	2	54–77	43–71	Moderate permissiveness
	3	78–85	72–83	Average scores
	4	86–94	84–93	Moderate restrictiveness
	5	95–110	97–117	Extreme restrictiveness

**Table 6:** Validity and reliability of the instruments used.

Instruments	Validity	Reliability
Parenting Styles and Dimensions Questionnaire	Content validity: Developed based on established theoretical frameworks of parenting styles. Empirically validated through expert review and pilot testing.	Internal consistency reliability: Cronbach’s alpha coefficient > 0.80 for all subscales. Test-retest reliability: Intraclass correlation coefficient (ICC) > 0.70.
Cognitive Skills Rating System Children	Content validity: Comprehensive assessment of various cognitive domains (e.g., verbal comprehension, perceptual reasoning) based on established theories of intelligence. Empirically validated through pilot studies.	Internal consistency reliability: Cronbach’s alpha > 0.90 for all subtests. Test-retest reliability: ICC > 0.85.
Social Skills Rating System Children	Content validity: Grounded in theories of social and emotional development. Items selected to assess various dimensions of social competence and behavior.	Internal consistency reliability: Cronbach’s alpha > 0.80 for all subscales. Test-retest reliability: ICC > 0.75.

**Table 7:** Descriptive data—first hypothesis.

Mother and Father Dyad	N	Mean	Std. Deviation	Std. Error	95% Confidence Interval for Mean		Min.	Max.
					Lower Bound	Upper Bound		
permissive–permissive	30	13.36	1.06	0.19	12.96	13.76	12	15
democratic–democratic	30	13.66	1.09	0.20	13.22	14.04	12	16
authoritarian–authoritarian	26	13.30	1.01	0.19	12.89	13.71	12	15
authoritarian–permissive	28	13.21	1.19	0.22	12.75	13.67	11	15
democratic–authoritarian	31	13.32	1.37	0.24	12.81	13.82	11	16
democratic–permissive	26	13.57	1.47	0.28	12.98	14.17	11	16
Total	171	13.40	1.20	0.09	13.22	13.58	11	16

**Table 8:** Descriptive data—second hypothesis.

Mother and Father Dyad	N	Mean	Std. Dev.	Std. Error	95% Confidence Interval for Mean		Minim	Maxim
					Lower Bound	Upper Bound		
permissive–permissive	30	12.90	0.95	0.17	12.54	13.25	11	15
democratic–democratic	30	14.66	0.47	0.08	14.48	14.84	14	15
authoritarian–authoritarian	26	12.15	0.88	0.17	11.79	12.50	11	14
authoritarian–permissive	28	11.85	0.97	0.18	11.48	12.23	11	14
democratic–authoritarian	31	12.77	0.80	0.14	12.47	13.06	11	14
democratic–permissive	26	13.53	1.24	0.24	13.03	14.03	11	15
Total	171	13.00	1.29	0.09	12.80	13.19	11	15

## Data Availability

Due to confidentiality agreements with the participants, this study’s data are available only upon request from the author.
